# Effects of Zn-Doped Mesoporous Bioactive Glass Nanoparticles in Etch-and-Rinse Adhesive on the Microtensile Bond Strength

**DOI:** 10.3390/nano10101943

**Published:** 2020-09-29

**Authors:** Yeonju Choi, Woogyeong Sun, Yeon Kim, In-Ryoung Kim, Mi-Kyung Gong, Seog-Young Yoon, Moon-Kyoung Bae, Bong-Soo Park, Soo-Byung Park, Yong-Il Kim

**Affiliations:** 1 Department of Orthodontics, Dental Research Institute, Pusan National University, Yangsan 50612, Korea; cdent1213@naver.com (Y.C.); mkgong10@gmail.com (M.-K.G.); sbypark@pusan.ac.kr (S.-B.P.); 2 School of Materials Science and Engineering, Pusan National University, Busan 46241, Korea; nonplayer64@pusan.ac.kr (W.S.); syy3@pusan.ac.kr (S.-Y.Y.); 3 Department of Oral Physiology, School of Dentistry, Pusan National University, Yangsan 50612, Korea; graceyeon88@gmail.com (Y.K.); mkbae@pusan.ac.kr (M.-K.B.); 4 Department of Oral Anatomy, School of Dentistry, Pusan National University, Yangsan 50612, Korea; biowool@pusan.ac.kr (I.-R.K.); parkbs@pusan.ac.kr (B.-S.P.); 5 Dental and Life Science Institute, Pusan National University, Yangsan 50612, Korea

**Keywords:** mesoporous bioactive glass nanoparticles, zinc, dental adhesives, matrix metalloproteinases, microtensile bond strength, human dental pulp stem cell

## Abstract

The purpose of this study was to assess the effects in the dentin bond strength of dental adhesives (DAs) and biological effects using zinc (Zn)-doped mesoporous bioactive glass nanoparticles (MBN-Zn). Synthesized MBN and MBN-Zn were characterized by scanning electron microscopy (SEM), X-ray diffraction and the Brunauer, Emmett and Teller (BET) method. The matrix metalloproteinases (MMP) inhibition effects of DA-MBN and DA-MBN-Zn were analyzed. The microtensile bond strength (MTBS) test was conducted before and after thermocycling to investigate the effects of MBN and MBN-Zn on the MTBS of DAs. The biological properties of DA-MBN and DA-MBN-Zn were analyzed with human dental pulp stem cells (hDPSCs). Compared with the DA, only the DA-1.0% MBN and DA-1.0% MBN-Zn exhibited a statistically significant decrease in MMP activity. The MTBS values after thermocycling were significantly increased in DA-1.0% MBN and DA-1.0% MBN-Zn compared with the DA (*p* < 0.05). It was confirmed via the MTT assay that there was no cytotoxicity for hDPSCs at 50% extract. In addition, significant increases in the alkaline phosphatase activity and Alizarin Red S staining were observed only in DA-1.0%MBN-Zn. These data suggest the 1.0% MBN and 1.0% MBN-Zn enhance the remineralization capability of DAs and stabilize the long-term MTBS of DAs by inhibiting MMPs.

## 1. Introduction

There has been remarkable improvement in dental adhesives (DAs) since 1955; however, the bond interface remains to be a very weak part of dental restoration. A bonding resin is restored to dentin by the formation of a hybrid layer [1,2,3]. Unfortunately, hydrolysis at the bond interface gradually reduces bond durability and stability in the hybrid layer [1,4,5,6,7,8]. Two major factors are involved in hydrolysis at the hybrid layer: (1) hydrolysis of polymers in the hybrid layer or the DA layer and (2) hydrolysis of collagen fibers exposed in the hybrid layer by matrix metalloproteinases (MMPs), which subsequently reduces the dentin–resin bond strength [9,10,11,12].

MMPs are host-derived proteolytic enzymes; they are calcium-dependent Zn-containing endopeptidases that degrade extracellular matrix (ECM) proteins [1,13,14,15,16,17]. Recent studies report that MMPs released by odontoblasts or ameloblasts [18,19] exist within the network of collagen fibers in the dentin in an inactivated state [1,20,21,22]. Inactivated MMPs are activated by proteinases, active oxygen, water, and low pH. Because activated MMPs degrade type I collagen fibers, which are a major component of the dentin matrix, researchers have claimed that MMPs have an important role in dentin–resin bond failures [1,20,21,22,23]. New strategies using MMP inhibitors have been developed to increase the longevity and stability of dentin–resin bonds.

The following MMP inhibitors were previously investigated: chemically modified tetracycline, doxycycline, and minocycline inhibit MMP-1, MMP-2, and MMP-12 and have been explored as MMP inhibitors for treating periodontal diseases [18,24,25]. Zoledronate, which is a type of bisphosphonate, inhibits the activation of MMPs by proteinases in dentin with cracks and delays tooth decay [18,24]. Epigallocatechin gallate detected in green tea inhibits the activity of MMP-2 and MMP-9 [18,24]. Ethylene diamine tetra-acetic acid (EDTA) inhibits MMP activity by chelating Zn or Ca ions [18,24,26]. The application of chlorohexidine (CHX) or galardin on acid-etched dentin reduces collagen degradation by MMPs and mitigates bond weakening by MMPs [14,18,27,28]. While these MMP inhibitors deliver significant efficacy in laboratory settings, issues arise during their clinical applications, such as the dilution of MMP inhibitors due to the moisture in the dentin and toxicity to the pulp [29]. Furthermore, research on the long-term effects of MMP inhibitors is lacking [29].

Zinc (Zn) is also an MMP inhibitor. Zn has antibacterial and osteogenic properties and has been extensively applied in dentistry [30]. A previous study reported that Zn significantly reduced MMP-mediated collagen degradation in dentin etched with phosphoric acid. This result showed that Zn inhibits MMPs by binding to the collagen-sensitive cleavage sites of MMPs [26].

To increase the long-term durability and bonding stability of dentin–resin adhesion, a drug delivery system (DDS), in which MMP inhibitors can be released for a long time, is required. Mesoporous bioactive glass nanoparticles (MBNs) are nano-size particles with small pores, which are employed for various purposes in the field of biomaterials. Owing to their stable structure, wide surface area, adsorbability, thermal stability, and chemical stability, MBNs are extensively used in biomedical applications. Furthermore, they have excellent ion releasing and remineralization properties [31,32].

Studies have been conducted by mixing the MMP inhibitor with the DAs. These studies have assessed the short-term reduction in collagen degradation by MMP inhibitors but have not sufficiently assessed their long-term effects and durability.

Thus, the purpose of this study was to evaluate the short- and long-term effects of DAs, including MBN and Zn-doped MBN (MBN-Zn), on the biological effects and dentin bond strength.

The null hypotheses to be tested are that the inclusion of MBN or MBN-Zn with in DAs (All bond universal; BISCO, Schaumburg, IL, USA) does not: (i) influence the microtensile bond strength of DAs (ii) induce the remineralization of DAs.

## 2. Materials and Methods

### 2.1. Synthesis of MBN and MBN-Zn

MBN and MBN-Zn were synthesized using a modified sol-gel method (Figure S1) [33]. Briefly, 10.15 g of calcium nitrate tetrahydrate, 8 mL of aqueous ammonia (Samchun, Pyeongtaek, Korea), 40 mL of 2-ethoxyethanol (Sigma-Aldrich, St. Louis, MO, USA), 80 mL of ethanol, and 4 g of hexadecyltrimethylammonium (CTAB; Sigma-Aldrich, St. Louis, MO, USA) were added to 600 mL of deionized water (DW). The mixture was stirred for 30 min. Next, 20 mL of tetraethyl orthosilicate (TEOS; Sigma-Aldrich, St. Louis, MO, USA) was added to the mixture. The mixture was stirred at room temperature for 30 min. Next, 1.89 mL of triethylphosphate (TEP; Sigma-Aldrich, St. Louis, MO, USA) was added to the mixture. The mixture was vigorously stirred at room temperature for 4 h. Once white precipitates were formed, they were washed with ethanol and dried in an oven at 60 °C for 24 h. Gel powder, which was dried for CTAB removal, was heated in a furnace at 600 °C for 5 h to perform calcination. The molar ratio (mol%) of SiO_2_:CaO:P_2_O_5_ was calculated to be 65:31:4 (Table 1).

To synthesize MBN-Zn, 11 g of calcium nitrate tetrahydrate, 8 mL of aqueous ammonia, 40 mL of 2-ethoxyethanol, 80 mL of ethanol, and 4 g of CTAB were added to 600 mL of DW. The mixture was stirred at room temperature for 30 min. A total of 20 mL of TEOS was added to the mixture. The mixture was stirred at room temperature for 30 min. Next, 2.04 mL of TEP was added, and the mixture was again stirred at room temperature for 30 min. Lastly, 2.23 g of Zn(NO_3_)_2_·6H_2_O (Sigma-Aldrich, St. Louis, MO, USA) was added, and the mixture was vigorously stirred at room temperature for 4 h. The remaining steps were identical to the steps in MBN synthesis. The molar ratio (mol%) of SiO_2_:CaO:P_2_O_5_:ZnO was calculated to be 60:31:4:5 (Table 1).

### 2.2. Characterisation of MBN and MBN-Zn

Scanning electron microscopy (SEM; SU-70, Hitachi, Tokyo, Japan) was used to examine the surface morphologies of MBN and MBN-Zn. Surface area and pore size were measured by N_2_ adsorption-desorption isotherms and the Brunauer-Emmett-Teller (BET) method (BELSORP-max, MicrotracBEL Corp., Osaka, Japan).

### 2.3. Fabrication of Experimental Samples

#### 2.3.1. Preparation of DA Mixed with MBN (DA-MBN) and DA Mixed with Zn-Doped MBN (DA-MBN-Zn)

MBN and MBN-Zn were prepared in three concentrations—0.1, 0.5, and 1.0 mass%—to add to DAs (All bond universal; BISCO, Schaumburg, IL, USA). DAs, zirconia balls, and MBN or MBN-Zn were added to a black e-tube to block light, and a rotating capsule mixing device (RotoMix, 3M ESPE, St. Paul, MN, USA) was utilised to mix the solution for 10 s.

#### 2.3.2. Resin Disk Preparation

To assess the mechanical properties of DA-MBN and DA-MBN-Zn, resin disks (diameter: 5 mm, height: 1 mm) were produced. DAs were injected into a brass mould, and a glass slide (width: 76 mm, length: 26 mm, and thickness: 2 mm) (MARIENFELD, Germany) was placed on top of the mould to create a flat surface. Next, light emitting diode (LED) light-curing units (VALO™ Cordless, >1000 mW/cm^2^, ULTRADENT Products, South Jordan, UT, USA) were used to photopolymerize from both the front and the back of the resin disks. The light irradiation part of the LED light-curing units was larger than or similar to the size of the resin disk, and was subjected to photopolymerization twice for 20 s with a thickness of 0.5 mm. Thereafter, for photopolymerization of the remaining monomers, photopolymerization was performed for 20 s on both the front and the back of the disks. The photopolymerization was performed at a position within 5 mm from the surface at 25 °C. The resin disks were sterilized with Ethylene oxide to conduct tests to assess the biological properties of DA-MBN and DA-MBN-Zn.

#### 2.3.3. Resin/Dentin Beam Preparation

A total of 40 caries-free premolars that were extracted from donors who gave informed consent were employed in the experiment. This study was reviewed and approved by The Institutional Review Board of Pusan National University Dental Hospital (PNUDH-2019-034). The teeth were stored in DW at 4 °C and applied within three months after receipt. The teeth were cut parallel to the occlusal surface using a low-speed diamond saw (Accutom-50, Struers Inc., Cleveland, OH, USA) with irrigation. The exposed surface was ground with 320- and 600-grit silicon carbide (SiC) paper with water irrigation. The teeth were randomly divided into seven groups (four teeth per group). The exposed surface was treated with 37% phosphoric acid for 15 s and washed. The remaining moisture was removed by air for 1~2 s avoiding chalky white surface. DAs were applied on the etched tooth surfaces, which were treated using a micro-brush for 10–15 s. The teeth were then dried for 10 s. This process of applying DAs and drying the teeth was repeated twice. The DAs were photopolymerized using LED light-curing units (VALO™ Cordless, Ultradent Products Inc., USA). Resin build-ups with a height of 2 mm were made with resin composites (Filtek Z-350XT, 3M ESPE, St. Paul, MN, USA), and the resin composites were photopolymerized using LED light-curing units for 20 s. The photopolymerization was performed at a position within 5 mm from the surface at 25 °C. This step was repeated to produce resin build-ups with a height of 4 mm. The teeth were stored in DW for 24 h and sectioned vertically with respect to the bonding interface into 1.0 × 1.0 × 8.0 mm resin/dentin beams.

### 2.4. Dissolution Test

Inductively-coupled plasma optical emission spectrometry (ICP-OES; Optima 8300, Perkin Elmer, USA) was implemented to assess the ion release capabilities of MBN and MBN-Zn using DA-MBN and DA-MBN-Zn disks (diameter: 5 mm, height: 1 mm) [34]. In accordance with the International Organization for Standardization (ISO 10993-12), the extraction ratio of resin disks (diameter: 5 mm, height: 1 mm) was 3 cm^2^/mL. And for ICP-OES test, the samples volume of 2 mL or more was required. So, the total of 14 resin disks were added to a tube that contains 2.56 mL of DW and precipitated at 37 °C for 1, 7, and 14 days [35]. The resin disks were removed, and the concentration of ions (calcium, phosphorus, silicon and Zn) released was measured.

### 2.5. Matrix Metalloproteinases (MMPs) Inhibition Test

A generic MMP Assay Kit (SensoLyte^®^ Generic MMP colorimetric assay kit-catalogue No. 72095, AnaSpec Inc., Lot# 131-029, Fremont, CA, USA) was used to examine the MMP inhibition effects of DA-MBN and DA-MBN-Zn in dentin tubules. The teeth were sectioned parallel to the occlusal surface using a low-speed diamond saw with irrigation to expose the mid-coronal dentin surface. The exposed surface was polished with 320, 600-grit SiC for 60 s to remove the smear layer. The teeth were stored in DW at 37 °C for 24 h and sectioned vertically with respect to the exposed surface into a total of 50 beams (1.0 × 1.0 × 4.0 mm). The dentin beams were treated with 37% phosphoric acid for 15 s and washed with DW for 15 s. The dentin beams were randomly divided into 10 groups (5 beams per group; Table S1; DW, DW + inhibitor, Ac (50%), Ac + DA, Ac + DA-0.1%MBN, Ac + DA-0.5%MBN, Ac + DA-1.0%MBN, Ac + DA-0.1%MBN-Zn, Ac + DA-0.5%MBN-Zn, Ac + DA-1.0%MBN-Zn). The dentins were immersed in 400 µL of DW (control), 400 µL of acetone (50%, acetone (Ac)/deionized water (DW) 1:1 vol%), 400 µL of DW/MMP inhibitor, or 400 µL of adhesive-acetone mix (1:1 vol%) and continuously stirred for 5 min. Next, the beams were immediately transferred to a 96-well plate that contains 250 μL of a generic MMPs substrate and treated at 37 °C for 2 h. After removing the beams from the plate, the total MMP activity was spectrophotometrically determined by measuring the absorbance of each well at 412 nm in a plate reader (Synergy HT microplate reader, BioTek Instruments, Winooski, VT, USA) [36].

### 2.6. Mechanical Properties

#### 2.6.1. Microtensile Bond Strength (MTBS) Test

The resin/dentin beams (15 beams per group for a total of 7 groups; DA, DA-0.1% MBN, DA-0.5% MBN, DA-1.0%MBN, DA-0.1% MBN-Zn, DA-0.5% MBN-Zn, DA-1.0% MBN-Zn) were fixed to a microtensile bond strength tester (MTBS tester; BISCO, Schaumburg, IL, USA) with self-adhesive resin cement (RelyX U200, 3M ESPE, USA). Tensile forces were applied at a cross-head speed of 1 mm/min until a beam fracture occurred [37].

#### 2.6.2. Thermocycling Test

The resin/dentin beams (15 beams per group for a total of 7 groups; DA, DA-0.1% MBN, DA-0.5% MBN, DA-1.0% MBN, DA-0.1% MBN-Zn, DA-0.5% MBN-Zn, DA-1.0% MBN-Zn) were placed in a thermocycling tester (TAEWONTECH, Seoul, Korea). The beams were immersed in 5 °C/55 °C water baths for 30 s each with a transfer time of 2 s between the two baths for a total of 5000 times. The thermocycling was followed by the MTBS test [38].

### 2.7. Biological Properties

#### 2.7.1. Cell Culture and MTT Assay

Human dental pulp stem cells (hDPSCs) were utilised to examine the biological properties of the DAs. hDPSCs were purchased from Lonza (Basel, Switzerland) and subcultured in Dulbecco’s modified Eagle’s medium (DMEM) that contains 10% foetal bovine serum (FBS; Gibco, NY, USA) 1% and antibiotics (Penicillin-streptomycin, Gibco, NY, USA) in a 5% CO_2_ incubator every 3 days. Cells from the 5th to the 7th generations were used in the experiment.

The 3-[4,5-dimethylthiazol-2-yl]-2,5-diphenyl tetrazolium bromide (MTT; Sigma, St. louis, MO, USA) colormetric assay was performed in accordance with ISO standards to assess the cytotoxicity of the resin disks constructed with DA-MBN and DA-MBN-Zn [39,40]. DA resin disks that did not contain MBN, DA-1.0% MBN resin disks with the highest MBN content, and DA-1.0% MBN-Zn resin disks with the highest MBN-Zn content, were employed.

The sterilized resin disks were added to a tube with DMEM and incubated at 37 °C in a 5% CO_2_ incubator for 24 h. A total of 5 resins were added per 1 mL of DMEM in accordance with ISO standards. The obtained original extract (100%) was diluted to 50% and 25% by mixing it with DMEM. hDPSCs were seeded at 1 × 10^4^/well in a 96-well culture plate and cultured using the 100, 50, and 25% extract DMEM for 24, 48, and 72 h, respectively. Pure DMEM (FBS 10%, penicillin 1%) was applied in the control group (0%). Following 24, 48, and 72 h of cell culture, the culture medium in each well was replaced with 100 μL of a fresh culture medium that contained 10 μL of MTT solution (5 mg/mL MTT in sterile PBS) and incubated at 37 °C in a 5% CO_2_ incubator for 4 h. The medium was then replaced with 100 μL of dimethyl sulfoxide (DMSO; Sigma, St. Louis, MO, USA) and stirred at room temperature for 10 min to produce formazan crystals. The crystals were dissolved to produce a purple color in acidified isopropanol. Absorbance was measured at 620 nm using a microplate reader (Sunrise, TECAN, Männedorf, Switzerland). The experiment was conducted in triplicate. The results of this experiment were expressed as relative cell viability (%) with respect to the control.

#### 2.7.2. Quantitative Assay of Alkaline Phosphatase (ALP) Activity

The resin disks were extracted from a differentiation medium (DM; composition: α-MEM, 10 mM β-glycerophosphate (Sigma, St. Louis, MO, USA), 50 μg/mL ascorbic acid (Sigma, St. Louis, MO, USA), 0.1 μM dexamethasone (Sigma, St. Louis, MO, USA)) for 24 h to induce differentiation. The obtained original extracts (100%) were diluted in DM to produce non-cytotoxic 25% extracts. hDPSCs were seeded at 1 × 10^5^/well in a 24-well plate. The medium was replaced with the 25% extract DM after 24 h. The medium was replaced every 2 days. To assess the odontogenic differentiation of hDPSCs, absorbance was measured at 405 nm using the alkaline phosphatase (ALP) detection kit (Sigma, St. Louis, MO, USA) according to the manufacturer’s protocol after 7 and 14 days of cell culture. The results were expressed in terms of the relative ALP activity with respect to the control.

#### 2.7.3. Alizarin Red S (ARS) Staining and Quantitative Detection

hDPSCs were seeded at 1 × 10^5^/well in a 24-well plate and cultured in a non-cytotoxic 25% extract DM. The medium was replaced every 2 days. After 7 and 14 days of cell culture, the cells were washed twice with phosphate-buffered saline (PBS) and fixed in 4% paraformaldehyde for 15 min. The cells were then washed three times with DW. Next, 500 µL of 1% Alizarin red S (Sigma, St. Louis, MO, USA) was added to each well to stain the cells at room temperature for 10 min. The cells were washed three times with DW. After drying the samples, images of the culture plate were obtained. Next, 250 µL of 10% acetic acid was added to each well to visualize and quantify staining. Absorbance was measured at 492 nm using a microplate reader (Sunrise, TECAN, Männedorf, Switzerland). The results were expressed in terms of the relative mineralization level with respect to the control group.

### 2.8. Non-Cellular Bioactivity of DA-MBN and DA-MBN-Zn

The resin disks were immersed in simulated body fluid (SBF) at 37 °C for 28 days to assess their non-cellular bioactivity. Images of resin disk surfaces were obtained using SEM (MIRA3, Tescan, Brno, Czech Republic) to examine the surface morphology of the resin disks before and after immersion in the SBF. X-ray diffraction (XRD) spectroscopy was performed using an Ultima IV multipurpose XRD system (Rigaku, The Woodland, TX, USA) at 40 kV and 40 mA, with a scanning speed of 0.1°/min, on the disk surfaces [40,41].

### 2.9. Statistical Analysis

The results were compared between the experimental groups using a one-way analysis of variance (ANOVA). Tukey’s HSD test was performed as a post-hoc test. All statistical analyses were performed using the SPSS program (IBM, Armonk, NY, USA).

## 3. Results

### 3.1. Characterisation of MBN and MBN-Zn

**Figure 1:**
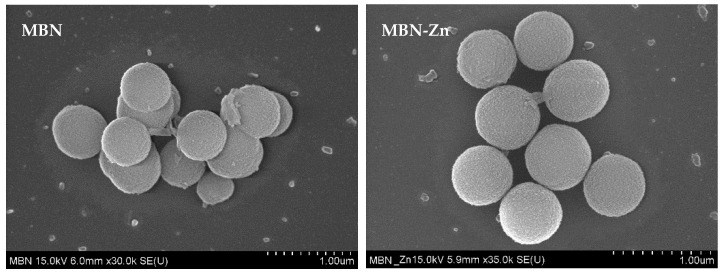
SEM images of MBN and MBN-Zn. Shape of the particles is spherical, and the diameter of the particles is approximately 300–600 nm (nano size). MBN-Zn: zinc-doped mesoporous bioactive glass nanoparticles.

The morphologies of MBN and MBN-Zn were examined using SEM (Figure 1). Both MBN and MBN-Zn featured diameters of approximately 300–600 nm and were globular.

**Figure 2:**
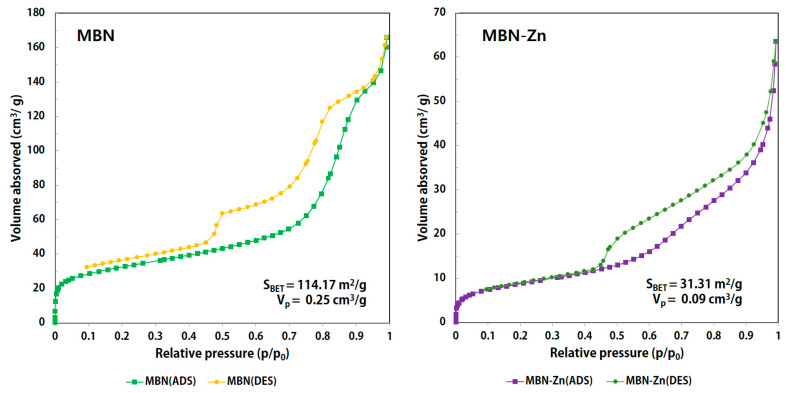
N_2_ adsorption-desorption isotherms of MBN and MBN-Zn. MBN and MBN-Zn represent the type-IV isotherm. The structure features a type-H1 hysteresis loop, which is related to mesoporous materials with approximately uniform pores.

Surface area and pore size were measured by N_2_ adsorption-desorption isotherms and the BET method. As shown in Figure 2, MBN and MBN-Zn represent a type-IV isotherm (exhibited characteristics of the type-IV isotherm of mesoporous materials according to the International Union of Pure and Applied Chemistry (IUPAC) classification). The structure features a type-H1 hysteresis loop, which is related to mesoporous materials with approximately uniform pores. The pore size distributions of MBN and MBN-Zn were narrow, in which most pore sizes were near the average sizes of 8.76 nm and 11.61 nm.

### 3.2. Characterisation of DA-MBN and DA-MBN-Zn

#### In-Vitro Dissolution Test

**Figure 3:**
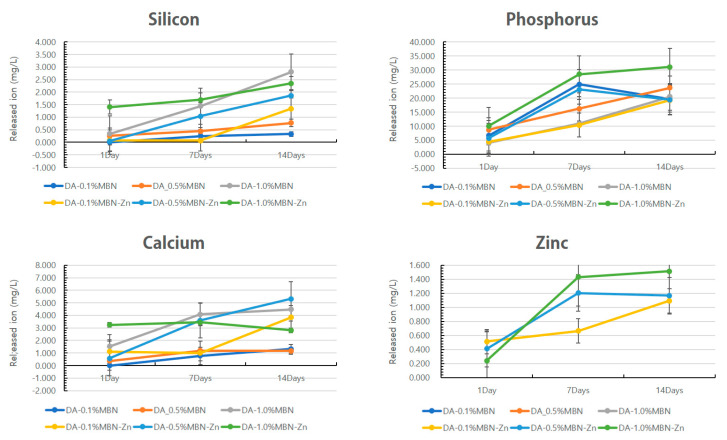
Change in silicon, phosphorus, calcium, Zn concentration over time. Ion elution amount of calcium in DA-1.0%MBN-Zn increased until 7 days and decreased. The concentrations of silicon, phosphorus, calcium, and Zn ions increased over time for all other groups. DA: dental adhesive.

The changes in the concentrations of ions released from the photopolymerized resin disks were shown in Figure 3. The concentration of calcium ion increased until 7 days and decreased in the DA-1.0% MBN-Zn group. The concentrations of silicon, phosphorus, calcium, and Zn ions increased over time for all other groups.

### 3.3. MMP Inhibition Test

**Figure 4:**
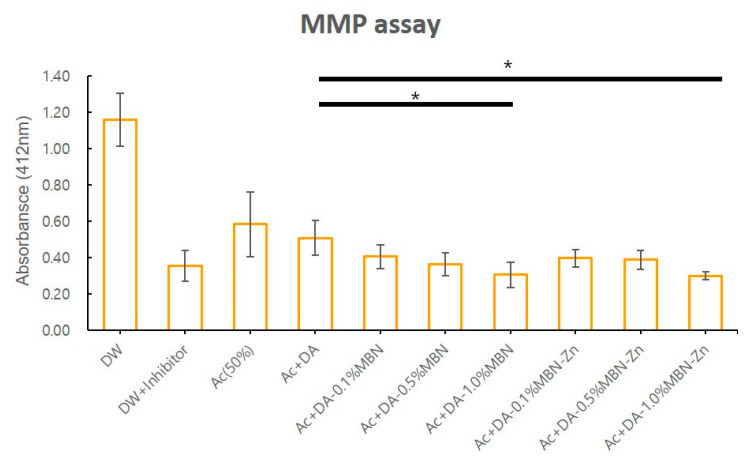
MMP activity after application of MBN or MBN-Zn mixed adhesives to human dentin. MMP activity was significantly decreased in DA-1.0%MBN and DA-1.0%MBN-Zn compared with that in the controls (DW, Ac (50%) and Ac + DA groups (Ac: solvent of dental adhesives)) after application of dental adhesives to dentin etched with 37% phosphoric acid (5 beams per group). * ANOVA was performed. The asterisks indicate that the *p*-value is significantly different (*p* < 0.05). Error bars indicate the ± standard deviation. MMP: matrix metalloproteinases; DW: deionized water; Ac: acetone.

The effect of the DA, DA-MBN and DA-MBN-Zn on endogenous MMP activity in human dentin was examined. Figure 4 shows the means and standard deviations of MMP activity (absorbance) in each group. The adhesives + acetone (solvent) significantly reduced the endogenous MMP activity within human dentin (*p* < 0.05). The MMP activity of the DA was compared between the DA-MBN group and DA-MBN-Zn group to investigate the effect of MBN or MBN-Zn on MMP activity. The DA-0.1% MBN, DA-0.5% MBN, DA-0.1% MBN-Zn and DA-0.5% MBN-Zn groups showed no statistically significant differences in MMP activity compared with the DA group (*p* > 0.05). The DA-1.0% MBN and DA-1.0% MBN-Zn groups showed a statistically significant reduction in MMP activity (*p* < 0.05) compared with the DA group (*p* < 0.05). However, no statistically significant differences in MMPs activity were observed between DA-1.0% MBN and DA-1.0% MBN-Zn.

### 3.4. Mechanical Properties

#### 3.4.1. MTBS Test

The MTBS test was performed to investigate the effect of MBN or MBN-Zn on the bonding ability of the DAs. Table 2 shows the test results of each group expressed in means and standard deviations. No statistically significant differences were obtained for the DA-MBN and DA-MBN-Zn groups compared with the control group (DA) (*p* > 0.05).

#### 3.4.2. MTBS Test after Thermocycling

The MTBS test was performed after treating the resin/dentin beams in a thermocycling tester to investigate the effect of MBN or MBN-Zn on the long-term bonding ability of the DAs. Table 2 shows the test results of each group expressed in means and standard deviations. No significant differences in MTBS values were detected for the DA-0.1% MBN, DA-0.5% MBN, DA-0.1% MBN-Zn, and DA-0.5% MBN-Zn groups compared with the control. (*p* > 0.05). A significant increase in MTBS values was observed for the DA-1.0% MBN and DA-1.0% MBN-Zn groups compared with the control (*p* < 0.05). However, no statistically significant differences in the MTBS values were obtained between DA-1.0% MBN and DA-1.0% MBN-Zn.

Mean MTBS values of DA, DA-0.1% MBN, DA-0.5% MBN, and DA-1.0% MBN groups; and DA-0.1% MBN-Zn, DA-0.5% MBN-Zn, DA-1.0% MBN-Zn groups were statistically similar to the control group (DA) (*p* > 0.05). After thermal aging, DA-1.0% MBN and DA-1.0% MBN-Zn showed higher mean MTBS values than the control group (DA) (*p* < 0.05).

### 3.5. Biological Properties

#### 3.5.1. MTT Assay

The cell viability of DA, DA-MBN, and DA-MBN-Zn for hDPSCs was analyzed using the MTT assay. An original extract (100%) prepared according to the ISO standards (10993-5-2009) was mixed with DMEM and diluted to 50 and 25% [35]. hDPSCs were cultured in the prepared media for 24, 48, and 72 h. Figure S2 shows the relative cell viability (%) of each group.

The relative cell viability decreased as the concentration of MBN and MBN-Zn increased in all groups after 24 h of cell culture. Relative cell viability was 100% or greater for all groups when the 25% extract was employed, which indicates that MBN and MBN-Zn were not cytotoxic. No statistically significant differences in relative cell viability were observed for DA-1.0% MBN and DA-1.0% MBN-Zn compared with DA when the 50% extract was employed.

Relative cell viability decreased as the concentration of MBN and MBN-Zn increased in all groups, except for DA-1.0% MBN-Zn after 48 h of cell culture. No statistically significant differences in relative cell viability were detected for all groups compared with the control when 50% and 25% extracts were applied.

No statistically significant differences in relative cell viability were obtained for all groups and extract concentrations compared with the control group, except for the DA-1.0% MBN group with 50% extract and the DA-1.0% MBN-Zn group with 25% extract after 72 h of cell culture. No statistically significant difference in relative cell viability was detected for the DA-1.0% MBN-Zn group compared with the control group when 25% extract was applied.

#### 3.5.2. ALP Activity in hDPSCs

**Figure 5:**
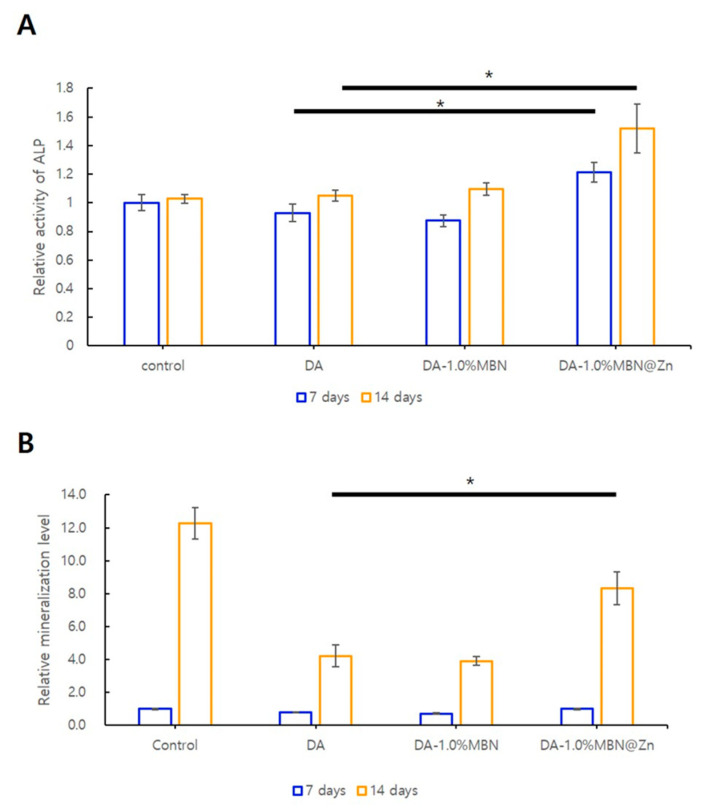
(**A**) Alkaline phosphatase (ALP) activity of hDPSC incubated on the surface of DA, DA-MBN and DA-MBN-Zn at 7 and 14 days. (**B**) Effect of DA-1.0% MBN and DA-1.0% MBN-Zn on mineralization of hDPSC for 7 and 14 days. ALP activity was significantly increased in DA-1.0% MBN-Zn compared with that in the control group (DA). Quantification of Alizarin Red S staining of hDPSC cultures. Alizarin Red S staining was significantly increased in DA-1.0% MBN-Zn compared with that in the control group (DA) at 14 days. * ANOVA was performed. The asterisks indicate that the *p*-value is significantly different (*p* < 0.05).

ALP activity was measured after 7 and 14 days of cell culture to evaluate the effect of DA, DA-MBN, and DA-MBN-Zn on odontogenic differentiation of hDPSCs (Figure 5). ALP activity was increased in the DA-1.0% MBN and DA-1.0% MBN-Zn compared with the control group (DA) after 7 and 14 days. A statistically significant increase in ALP activity was only observed in the DA-1.0% MBN-Zn group.

#### 3.5.3. ARS Staining

Alizarin Red S staining was performed to examine the effect of DA, DA-MBN and DA-MBN-Zn on the mineralization potential of DAs in hDPSCs (Figure 5). Following staining quantification, no statistically significant differences in staining were observed for DA-MBN and DA-MBN-Zn compared with DA after 7 days. A statistically significant increase in staining was observed for DA-1.0% MBN-Zn compared with the control group (DA) after 14 days. This result indicated that MBN-Zn increased the mineralization potential of the DA in hDPSCs.

### 3.6. Non-Cellular Bioactivity of DA-MBN and DA-MBN-Zn

#### 3.6.1. SEM after SBF Soaking

**Figure 6:**
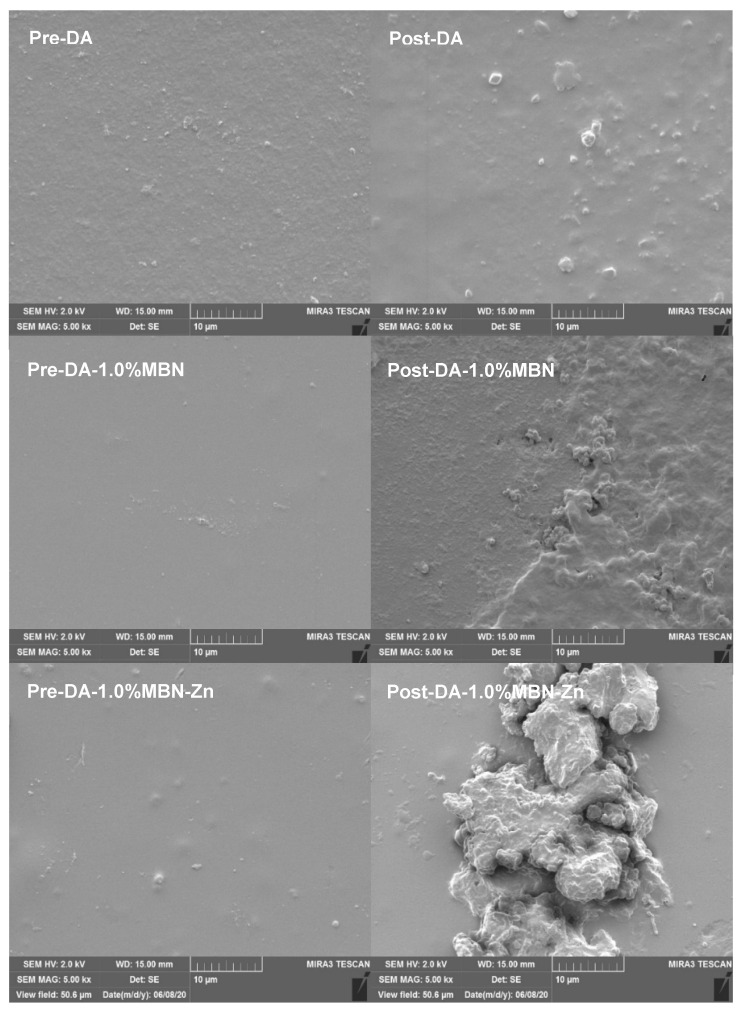
Non-cellular bioactivity of resin disk before and after immersing in the simulated body fluid (SBF) at 37 °C for 28 days as determined using SEM. After immersing in the SBF for 28 days at 37 °C, apatite-like crystallites were observed on the surface of all groups. The shape of the apatite that formed on the surfaces of the DA-MBN-Zn resin disks is substantially larger than other resin disks in the SBF.

Before and after immersing the resin disks constructed of DA, DA-1.0% MBN, and DA-1.0% MBN-Zn in the SBF at 37 °C for 28 days, the surface morphology was observed by SEM, and an apatite was observed on the surface of DA, DA-1% MBN and DA-1% MBN-Zn resin disks (Figure 6). The apatite that formed on the surfaces of the DA-1% MBN-Zn resin disks were considerably larger than other crystallites.

#### 3.6.2. XRD after SBF Soaking

**Figure 7:**
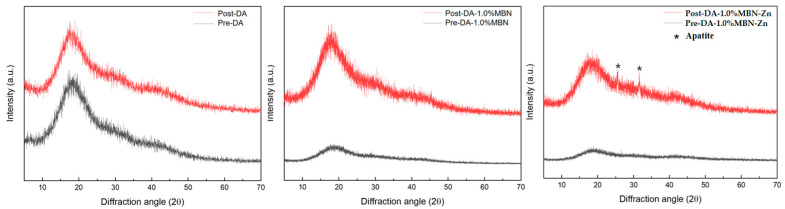
XRD spectra of DA (control group), DA-1.0% MBN and DA-1.0%MBN-Zn resin disks before and after immersion in the SBF at 37 °C for 28 days. After immersing the resin disks in the SBF at 37 °C for 28 days, an apatite peak (JCPDS #9-0432) was observed on DA-1.0%MBN-Zn.

Figure 7 shows the XRD patterns of the DA (control group), DA-1.0% MBN and DA-1.0% MBN-Zn resin disks before and after immersion in the SBF at 37 °C for 28 days. After immersing the resin disks in the SBF at 37°C for 28 days, an apatite peak (JCPDS #9-0432) was observed on DA-1.0% MBN-Zn.

## 4. Discussion

MMP inhibitors showed significant efficacy in laboratory settings but exhibited a reduction in the concentration of MMP inhibitors owing to the moisture from dentin in clinical settings [29]. Studies have been conducted by mixing the MMP inhibitors with the DAs. These studies assessed the short-term effects but did not sufficiently assess their long-term effects and durability. In this study, Zn, as an MMP inhibitor, was added to MBN, which can release ions for a long period to synthesize MBN-Zn, and the short- and long-term effects of MBN-Zn on the dentin–resin bond strength were examined.

In this study, All Bond Universal^®^ (DA; BISCO, Schaumburg, IL, USA), which did not contain a filler, was employed as a DA. DA is a universal adhesives system that can be employed as self-etch adhesives and etch-and-rinse adhesives [42,43]. Munoz M.A. et al. reported that DA applied in the etch-and-rinse strategy had high immediate MTBS values unlike those of other universal adhesives and DAs applied in the self-etch strategy, but their MTBS values decreased after six months [44]. This finding indicates that DA employed in the etch-and-rinse strategy has lower long-term dentin–resin bonding stability. Thus, the DA utilised in the etch-and-rinse strategy was more suitable for examining the long-term dentin–resin bonding ability of MBN-Zn.

To synthesize porous MBN and MBN-Zn with large surface areas, high stability, and excellent remineralization and ion release capabilities, the modified sol-gel method was utilised [33]. As shown in the SEM images in Figure 1, the synthesized MBN and MBN-Zn were nanosized globular particles. The surface area and pore size of the particles were measured using N_2_ adsorption-desorption isotherms and the BET method. Both MBN and MBN-Zn were type-IV isotherms and exhibited a type-H1 hysteresis loop. These characteristics are observed in porous substances with approximately uniform pores. The surface areas of particles were also increased owing to their porous structures (Figure 2).

We investigated whether the MBN-Zn particles would induce the release of Zn^2+^ within the DA. As shown in Figure 3, the concentrations of the released Si^4+^, PO_4_^3−^, Ca^2+^, and Zn^2+^ increased over time in almost all the groups. In the DA-1.0% MBN-Zn group, the concentration of the released Ca^2+^ increased until 7 days and subsequently decreased as Zn^2+^ binds with PO_4_^3−^ and Ca^2+^ to form calcium Zn phosphate in an aqueous solution [45].

The MMP inhibition effect of Zn^2+^ released from DA-MBN-Zn was analyzed using a generic MMP assay. No statistically significant difference in MMP activity was observed for groups with low concentrations of MBN and MBN-Zn, including the DA-0.1% and 0.5% MBN groups and the DA-0.1% and 0.5% MBN-Zn groups compared with the control group (DA). In contrast, a statistically significant reduction in MMP activity was detected for groups with relatively high concentrations of MBN and MBN-Zn, including the DA-1.0% MBN and DA-1.0% MBN-Zn groups compared with the control group (i.e., DA) (Figure 4). These results indicate that MMP activity is decreased by not only Zn^2+^ but also MBN. Previous studies of the mechanisms of MBN-reducing MMP activity have been reported [46]. The results of these studies are summarised as follows: 1) Ions released from MBN bind to a specific region of the exposed collagen fibers to change their three-dimensional structures, which disables the binding of the collagen fibers to the sensitive cleavage sites of MMPs [23]; 2) Electrostatic interactions occur between MMP-1 or MMP-9 and calcium phosphate (CaP) precipitates that form aggregates with a large molecular size and low mobility, which reduces the enzymatic activity [47]; 3) HAs inactivate MMP-1 and MMP-3 within bones [48]; and 4) Ca^2+^ and Na^+^ locally induce alkalization to reduce the MMP activity that generally occurs in acidic environments [49,50,51]. In contrast, MMP-mediated collagen degradation activity depends on the concentrations of calcium and Zn, but a relatively high concentration of Zn ions interferes with the MMP-mediated collagen degradation activity. Osorio et al. reported that high concentrations of Zn significantly reduce collagen fiber degradation by MMPs [23]. In this study, a similar reduction in MMP activity was observed for DA-1.0% MBN and DA-1.0% MBN-Zn. In a previous study, Zn chloride (3.33 mg/mL) mixed with artificial saliva significantly reduced collagen fiber degradation in a dose-dose dependent manner [18]. In another study, Zn nitrate powder (5 wt%) added to self-etch adhesives reduced the MTBS values [52]. Thus, the inhibitory effect of Zn on endogenous MMPs can vary depending on the Zn concentration, type of Zn chemicals, and substances to which Zn is added. In this study, the concentration of Zn released from DA-1.0% MBN-Zn was less than 2.0 ppm (0.002 mg/mL). The similar reduction in MMP activity by DA-1.0% MBN and DA-1.0% MBN-Zn may be attributed to the concentration because the concentration of Zn^2+^ released from DA-MBN-Zn was too low to significantly reduce MMP activity.

The remineralization of the hybrid layer against collagen desalted by acidic substances affects the long-term stability of DAs [53]. A previous study discovered that DAs with remineralization properties release ions, such as Ca^2+^ and P^3-^, to autonomously heal microleakages at the resin-dentin bonding interface and neutralize pH to increase the long-term bonding ability of resin restorations [54]. Additionally, these DAs promote the formation of HA crystals in partially demineralized dentin [39]. This mechanism remineralizes the demineralized regions within the hybrid layer to increase the long-term stability of DAs [39]. Kavrik reported that MBN induces biomimetic remineralization as a source of CaP [55]. The DA-MBN and DA-MBN-Zn utilized in this study release Ca^2+^ and PO_4_^3−^ and may induce biomimetic remineralization (Figure S3 and Table S2). As HA is mineralized, endogenous MMPs within dentin become fossilized and inactivated. Consequently, collagen fiber do not become degraded. To investigate whether this remineralization process is also induced by DA-MBN and DA-MBN-Zn, resin disks were immersed in the SBF at 37 °C for 28 days, and the surface morphology and composition were analyzed using SEM and XRD. Figure 6 shows HA-like crystallites on the surface of all groups. These crystallites were formed by PO_4_^3−^, Ca^2+^ on the surface of both resin disks. However, apatite-like minerals of DA-1.0%MBN-Zn were considerably larger than those of DA and DA-1% MBN. An apatite peak (JCPDS #9-0432) was only observed on DA-1.0% MBN-Zn as Zn^2+^ binds with PO_4_^3−^ and Ca^2+^ to form calcium Zn phosphate in an aqueous solution. It could be assumed that Zn^2+^ facilitates the formation of a ZnO rich layer that will permit Ca and P deposits and further remineralization [45].

According to a previous study, the interaction effect between the monomers and additives eluted from the light-cured resin can affect the cytotoxicity and induction of DNA double-strand breaks (DNA-DSBs) [56]. Therefore, in this study, MTT assay was performed to evaluate the cytotoxicity of the experimental group. The resin disk used in this experiment was made 1mm thickness and light irradiation was conducted for 20 s so as not to cause the related genotoxic effect [57]. Based on MTT assay result, the 50% extract was employed to analyze the effects of DA, DA-MBN and DA-MBN-Zn on the odontogenic differentiation and mineralization potential of hDPSCs (Figure 5). A significant increase in the ALP activity and Alizarin Red S staining was observed only for DA-1.0% MBN-Zn compared with DA. This result verified that DA-1.0% MBN-Zn or higher concentrations can induce odontogenic differentiation and mineralization in hDPSCs.

MTBS tests indicated that the addition of MBN or MBN-Zn did not affect the immediate bond strength of the DA. To examine the long-term effects of MBN and Zn^2+^ on bond strength, resin/dentin beams were aged by a thermocycling machine. Saboi et al. reported that extensive thermocycling (6000 cycles) reduces the bond strength of etch-and-rinse adhesives [52]. Another study reported that 5000 cycles of thermocycling drastically reduced the bond strength of etch-and-rinse and self-etch adhesives [58]. These findings conclude that many cycles of thermocycling reduce the bond strength of DAs [59]. In this study, the MTBS values after thermocycling significantly increased in the DA-1.0% MBN and DA-1.0% MBN-Zn groups compared with the control group (DA) (*p* < 0.05). This increase showed that inhibition of MMP by DA 1.0% MBN and DA-1.0% MBN-Zn did not increase the immediate binding strength but contributed to the long-term stabilization of the binding strength of DA. There have been studies of dose response between Zn concentration and MMP inhibition. de Souza et al. reported that the concentration of physiological Zn inhibited soluble MMP-2 by 52% and MMP-9 by 40% [60]. When the Zn concentration was doubled to 30 M, the inhibition rate increased from 52% to 64.3% [61]. To more clearly understand the MMP inhibition effect of Zn in DAs applied in the etch-and-rinse strategy, additional experiments must be performed using MBN-Zn with concentrations greater than 1.0% or a higher ratio of Zn.

In this study, MBN and MBN-Zn enhanced the remineralization capability of the DAs and stabilized the long-term MTBS of the DAs by acting as MMP inhibitors. Additionally, DA-MBN-Zn increased Zn^2+^ release in DW over time. However, no significant reduction in endogenous MMP activity and changes in MTBS values following thermocycling were observed between DA-MBN and DA-MBN-Zn. A similar reduction in MMP activity and an increase in MTBS values after thermocycling were observed for MBN and MBN-Zn. This reduction may be attributed to the concentration because the concentration of Zn released from DA-1.0% MBN-Zn with the highest MBN-Zn content was not sufficient to drastically reduce MMP activity.

## 5. Conclusions

In this study, the 1.0% MBN and 1.0% MBN-Zn enhance the remineralization capability of DAs and stabilize the long-term MTBS of DAs by inhibiting MMPs. When the concentration of Zn released from MBN-Zn is less than 2.00 ppm, Zn cannot sufficiently inhibit MMPs and increase the bond strength.

## Figures and Tables

**Table 1:** Compositions of MBN and MBN-Zn.

	Composition (mol%)			
	SiO_2_	CaO	P_2_O_5_	ZnO
MBN	65	31	4	0
MBN-Zn	60	31	4	5

**Table 2:** Mean microtensile bond strength values and mean microtensile bond strength values after thermocycling after MBN or MBN-Zn mixed adhesives application to human dentin.

Group	n	MTBS	MTBS after Thermocycling
DA	15	36.59 ± 10.66	28.77 ± 4.16
DA-0.1% MBN	15	36.83 ± 10.18	35.66 ± 4.35
DA-0.5% MBN	15	37.59 ± 11.31	36.98 ± 5.30
DA-1.0% MBN	15	37.64 ± 8.11	37.57 ± 10.29 *
DA-0.1% MBN-Zn	15	36.25 ± 7.50	35.59 ± 10.02
DA-0.5% MBN-Zn	15	37.02 ± 8.89	35.81 ± 11.35
DA-1.0% MBN-Zn	15	38.39 ± 8.09	37.85 ± 7.12 *

* ANOVA was performed. The asterisks indicate that the *p*-value is significantly different (*p* < 0.05).

## Supplementary Materials

The following materials are available online at https://www.mdpi.com/2079-4991/10/10/1943/s1: Figure S1: Flowchart of MBN and Zn-doped MBN synthesis, Figure S2: Cell viability analysis; hDPSCs were incubated on DA, DA-1.0% MBN, and DA-1.0% MBN at 100, 50 and 25% extract, respectively, for 24, 48, and 72 h, Figure S3: FT-IR spectrum for DA, DA-1.0% MBN, and DA-1.0% MBN-Zn, Table S1: Matrix of metalloproteinase (MMPs) inhibition; test samples, Table S2: Assignments of FT-IR peaks for DA, DA-1.0% MBN, and DA-1.0% MBN-Zn.
