# Supplementary material for: Effects of Zn-Doped Mesoporous Bioactive Glass Nanoparticles in Etch-and-Rinse Adhesive on the Microtensile Bond Strength
Source: Nanomaterials (Basel). 2020 Sep 29;10(10):1943. doi: 10.3390/nano10101943 (PMC7601785; doi:10.3390/nano10101943)
Supplement: Supplementary file 1 [file nanomaterials-10-01943-s001.pdf]

nanomaterials

Supplementary Materials

Effects of Zn-Doped Mesoporous Bioactive Glass Nanoparticles in Etch-and-Rinse Adhesive on the Microtensile Bond Strength

Yeonju Choi [1], Woogyeong Sun [2], Yeon Kim [3], In-Ryoung Kim [4], Mi-Kyung Gong [1], Seog-Young Yoon [2], Moon-Kyoung Bae [3], Bong-Soo Park [4], Soo-Byung Park [1] and Yong-Il Kim [1,5,*]

[1] Department of Orthodontics, Dental Research Institute, Pusan National University, Yangsan 50612, Korea; cdent1213@naver.com (Y.C.); mkgong10@gmail.com (M.-K.G.); sbypark@pusan.ac.kr (S.-B.P.)
[2] School of Materials Science and Engineering, Pusan National University, Busan 46241, Korea; nonplayer64@pusan.ac.kr (W.S.); syy3@pusan.ac.kr (S.-Y.Y.)
[3] Department of Oral Physiology, School of dentistry, Pusan National University, Yangsan 50612, Korea; graceyeon88@gmail.com (Y.K.); mkbae@pusan.ac.kr (M.-K.B.)
[4] Department of Oral Anatomy, School of dentistry, Pusan National University, Yangsan 50612, Korea; biowool@pusan.ac.kr (I.-R.K.); parkbs@pusan.ac.kr (B.-S.P.)
[5] Dental and Life Science Institute, Pusan National University, Yangsan 50612, Korea;
* Correspondence: kimyongil@pusan.ac.kr; Tel.: +82-55-360-5163

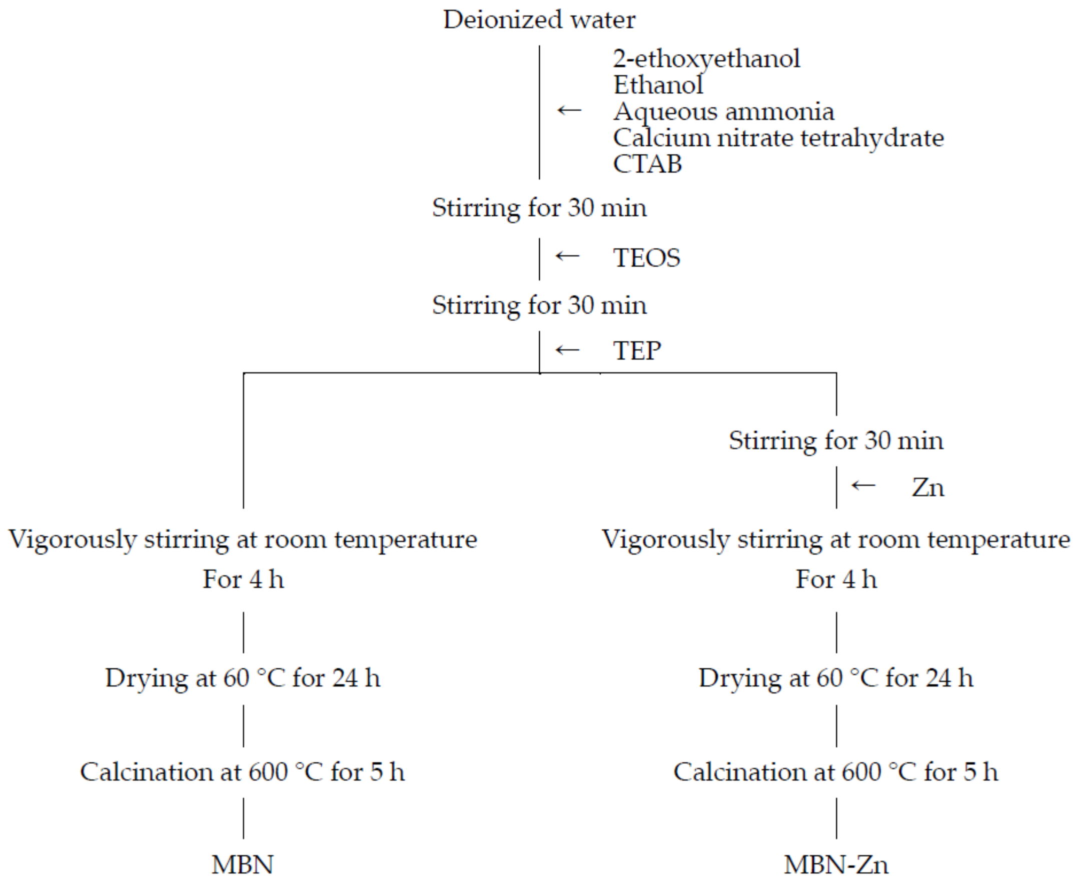

Figure S1. Flowchart of MBN and Zn-doped MBN (MBN-Zn) synthesis.

CTAB, cetyl trimethyl ammonium bromide; TEOS, tetraethyl orthosilicate; TEP, triethyl phosphate; MBN, mesoporous bioactive glass nanoparticle; MBN-Zn, Zn-doped MBN.

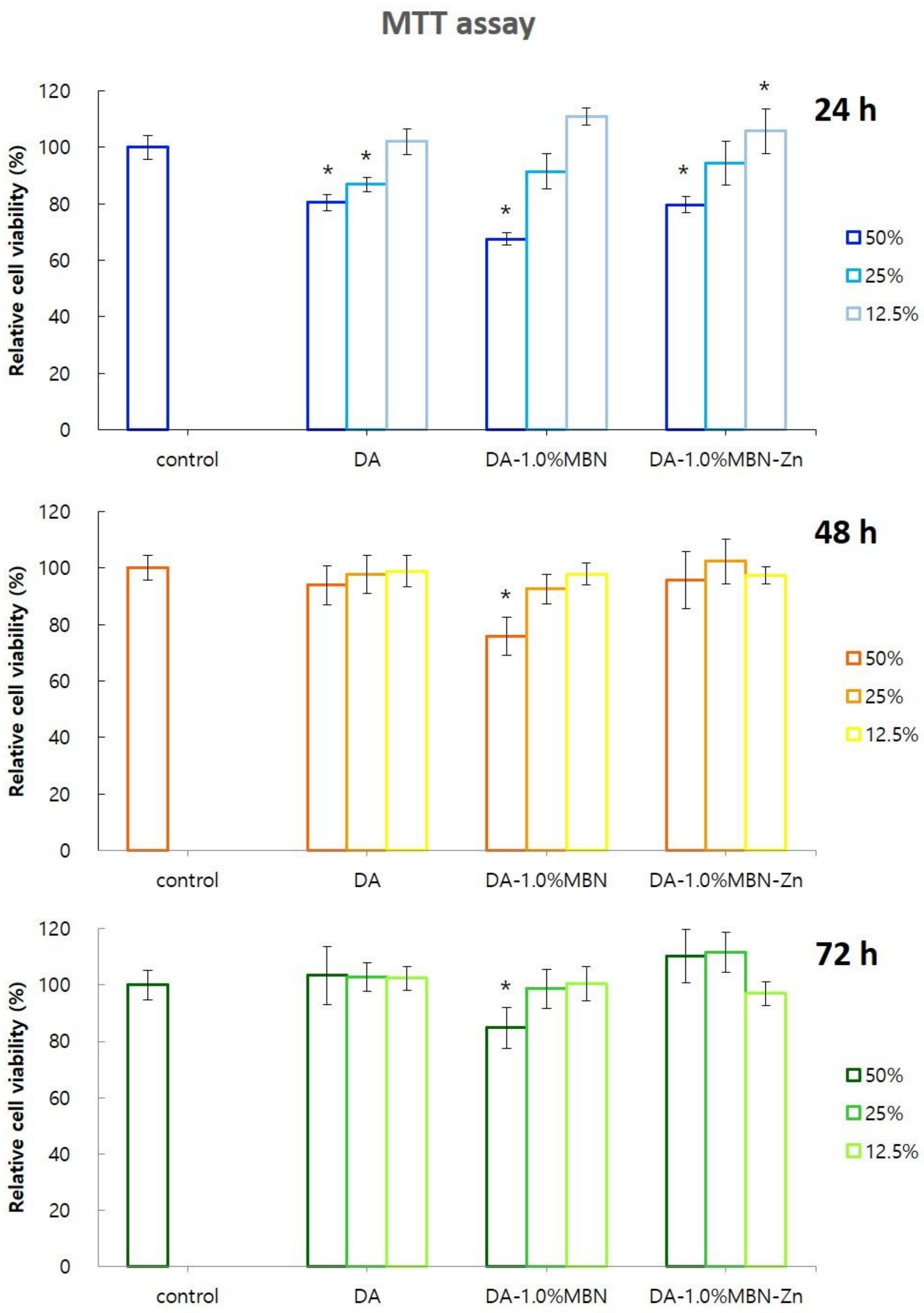

Figure S2. Cell viability analysis; hDPSCs were incubated on DA, DA-1.0%MBN, and DA-1.0%MBN at 100, 50 and 25% extract, respectively, for 24, 48, and 72 h. The cell viability with 50% extract was statistically similar to that of the control group. *ANOVA was performed and indicate that the *p*-value is not significantly different ($p < 0.05$). The error bars indicate the ± standard deviation.

Table S1. Matrix of metalloproteinase (MMP) inhibition; tested samples.

Groups		Composition	MMPs Substrate
DW		DW 400 μL	250 μL
DW+MMP inhibitor		DW 400 μL	250 μL + MMP inhibitor 100 μL
Ac 50%		DW 200 μL + Ac 200 μL	250 μL
Dental adhesive + Ac (1:1 vol%)	Ac + DA	Ac 200 μl + DA 200 μL	250 μL
	Ac + DA-0.1%MBN	Ac 200 μL + DA-0.1%MBN 200 μL	250 μL
	Ac + DA-0.5%MBN	Ac 200 μL + DA-0.5%MBN 200 μL	250 μL
	Ac + DA-1.0%MBN	Ac 200 μL + DA-1.0%MBN 200 μL	250 μL
	Ac + DA-0.1%MBN-Zn	Ac 200 μL + DA-0.1% MBN-Zn 200 μL	250 μL
	Ac + DA-0.5% MBN-Zn	Ac 200 μL + DA-0.5% MBN-Zn 200 μL	250 μL
	Ac + DA-1.0% MBN-Zn	Ac 200 μl + DA-1.0% MBN-Zn 200 μL	250 μL

Abbreviations: MMPs, matrix metalloproteinases; DW, deionized water; Ac, acetone; MBN, mesoporous bioactive glass nanoparticle; MBN-Zn, Zn-doped mesoporous bioactive glass nanoparticle; DA-0.1%MBN, 0.1% MBN mixed dental adhesives; DA-0.5%MBN, 0.5% MBN mixed dental adhesives; DA-1.0%MBN, 1.0% MBN mixed dental adhesives; DA-0.1%MBN-Zn, 0.1% MBN-Zn mixed dental adhesives; DA-0.5% MBN-Zn, 0.5% MBN-Zn mixed dental adhesives; DA-1.0% MBN-Zn, 1.0% MBN-Zn mixed dental adhesives.

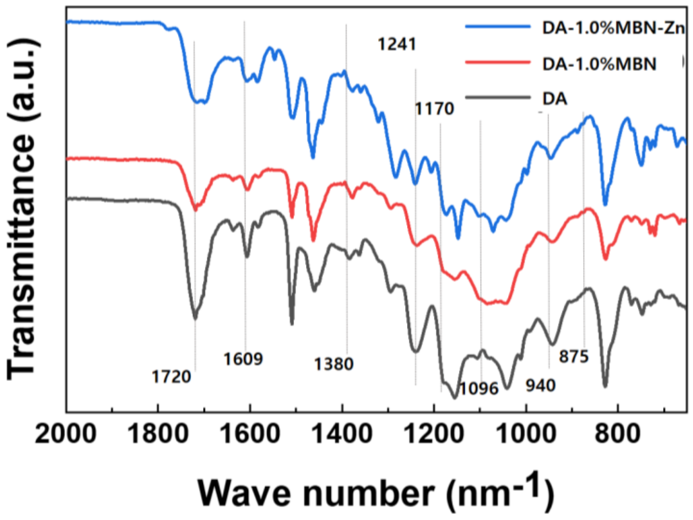

Figure s3. FT-IR spectrum for DA, DA-1.0%MBN and DA-1.0%MBN-Zn.

Table S2. Assignments of FT-IR peaks for DA, DA-1.0%MBN, DA-1.0%MBN-Zn.

**Wavenumber (cm^{-1})**	**Functional Group Assignment**
875	related to the presence of CO^{3-}
940	C=C stretching bend
1044	Si-O-Si stretching mode of vibration
1020-1110	Si-O-Si assymetric stretching vibration
1380	CH3 derfomation
1460	related to the presence of CO^{3-}
1545	C-N amide stretching
1609	CH2=CH stretching vibration
1720	C=O stretching
1170, 1241	Coupling between OH and CO of OH bending and CO stretching of neighboring carboxylic groups
